# 117 Allocation of Therapist Time: Mobilization of CFU’s in Hand w/Burn Injury

**DOI:** 10.1093/jbcr/irad045.090

**Published:** 2023-05-15

**Authors:** Sandra Fletchall, Grace Hartl

**Affiliations:** Rehabilitation Strategies 4 Catastrophic Injuries, LLC, Millington, Tennessee; 0x, Schenectady, New York; Rehabilitation Strategies 4 Catastrophic Injuries, LLC, Millington, Tennessee; 0x, Schenectady, New York

## Abstract

**Introduction:**

Movement of the hands and digits are used to perform communication and activities for IADL’s and competitive employment. Following a second or third degree to the hand(s), there is a potential for the development of burn scar contracture(s) (BSC), which decreases hand skills creating the potential for decreased quality of life.

The ABA data base identifies that 70% of burns are sustained to the upper torso, UE, hands. One ABA verified burn center created a study to identify the time required for the burn therapist to elongate hand CFU’s to pre-burn movement.

**Methods:**

The one-year retrospective study included unilateral and bilateral hand burns with LOS of 2 days or greater or had an outpatient OT session within one week of the burn.

Co-author created the Passive Motion Scoring Grid (PMSG), using a number score with higher number indicating greater passive movement. Education was provided to the OT’s to complete the PMSG at the beginning and end of each hand CFU elongation session. Otherwise, there was no change in the OT burn hand therapy treatment. The study included (5) OT’s with mean of 6 years of experience (3-46).

**Results:**

There were 50 patients, average age was 48.91 years with majority male and African American. All burns were secondary to thermal with burns of 2^nd^ and 3^rd^ degree.

There were 33 unilateral hand burns with an average TBSA of 7.02% and 17 patients sustained bilateral hand burns with an average TBSA of 21.3%.

Unilateral hand burns had an average number of CFU’s of 39.4 (11-68) and hand CFU’s accounted for 87.1% of the CFU’s. Average number of CFU’s in bilateral hand burns was 90.3 (8-196) with hand CFU’s accounting of 83.4% of the CFU’s.

A total of 539 OT sessions were provided to those with unilateral hand burns with an average of 18 OT sessions (3-36). Bilateral hand burns received a total of 487 OT sessions with average of 24.4 OT sessions (4-67)

Therapist time for hand CFU elongation to pre-burn status: unilateral hand burns, 43.5 minutes (SD=24.7) and bilateral hand burns was 53.5 minutes (SD=32.2). The minutes only reflect CFU elongation not the entire therapy time.

**Conclusions:**

Hand burns result in a small number for TBSA, however it results in a greater number of CFU’s than other body locati8ons. The greater number of CFU’s involved, indicates greater potential for development of BSC. At the completion of the burn OT therapy, no patient exhibited BSC.

Providing education and information to funding sources regarding CFU’s in hand burns, equating that to potential BSC development and time required for therapist direct hands-on techniques to obtain pre-burn movement can positively influence authorization for therapy.

**Applicability of Research to Practice:**

Can assist with allocating therapist time with hand burns. Can positively influence funding authorization for therapy.

